# Association of Expanded Prenatal Care Coverage for Immigrant Women With Postpartum Contraception and Short Interpregnancy Interval Births

**DOI:** 10.1001/jamanetworkopen.2021.18912

**Published:** 2021-08-02

**Authors:** Maria I. Rodriguez, Menolly Kaufman, Stephan Lindner, Aaron B. Caughey, Ana Lopez DeFede, K. John McConnell

**Affiliations:** 1 Department of Obstetrics and Gynecology, Oregon Health & Science University, Portland; 2 Center for Health Systems Effectiveness, Oregon Health & Science University, Portland; 3 Institute for Families in Society, University of South Carolina, Columbia

## Abstract

**Importance:**

Access to prenatal and postpartum care is restricted among women with low income who are recent or undocumented immigrants enrolled in Emergency Medicaid.

**Objective:**

To examine the association of extending prenatal care coverage to Emergency Medicaid enrollees with postpartum contraception and short interpregnancy interval births.

**Design, Setting, and Participants:**

This cohort study used a difference-in-differences design to compare the staggered rollout of prenatal care in Oregon with South Carolina, a state that does not cover prenatal or postpartum care. Linked Medicaid claims and birth certificate data from 2010 to 2016 were examined for an association between prenatal care coverage for women whose births were covered by Emergency Medicaid and subsequent short IPI births. Additional maternal and infant health outcomes were also examined, including postpartum contraceptive use, preterm birth, and neonatal intensive care unit admission. The association between the policy change and measures of policy implementation (number of prenatal visits) and quality of care (receipt of 8 guideline-based screenings) was also analyzed. Statistical analysis was performed from August 2020 to March 2021.

**Exposures:**

Medicaid coverage of prenatal care.

**Main Outcomes and Measures:**

Postpartum contraceptive use, defined as receipt of any contraceptive method within 60 days of delivery; short IPI births, defined as occurring within 18 months of a previous pregnancy.

**Results:**

The study population consisted of 26 586 births to women enrolled in Emergency Medicaid in Oregon and South Carolina. Among these women, 14 749 (55.5%) were aged 25 to 35 years, 25 894 (97.4%) were Black, Hispanic, Native American, Alaskan, Pacific Islander, or Asian women or women with unknown race/ethnicity, and 17 905 (67.3%) lived in areas with urban zip codes. Coverage of prenatal care for women in Emergency Medicaid was associated with significant increases in mean (SD) prenatal visits (increase of 10.3 [0.9] prenatal visits) and prenatal quality. Prenatal care screenings (eg, anemia screening: increase of 65.7 percentage points [95% CI, 54.2 to 77.1 percentage points]) and vaccinations (eg, influenza vaccination: increase of 31.9 percentage points [95% CI, 27.4 to 36.3 percentage points]) increased significantly following the policy change. Although postpartum contraceptive use increased following prenatal care expansion (increase of 1.5 percentage points [95% CI, 0.4 to 2.6 percentage points]), the policy change was not associated with a reduction in short IPI births (−4.5 percentage points [95% CI, −9.5 to 0.5 percentage points), preterm births (−0.6 percentage points [95% CI, –3.2 to 2.0 percentage points]), or neonatal intensive care unit admissions (increase of 0.8 percentage points [95% CI, −2.0 to 3.6 percentage points]).

**Conclusions and Relevance:**

This study found that expanding Emergency Medicaid benefits to include prenatal care significantly improved receipt of guideline-concordant prenatal care. Prenatal care coverage alone was not associated with a meaningful increase in postpartum contraception or a reduction in subsequent short IPI births.

## Key Points

**Question:**

When prenatal care coverage is an included benefit in Emergency Medicaid—a program of restricted Medicaid services for recent immigrants who have low income and are pregnant—is there an improvement in postpartum contraception and a subsequent reduction in short interpregnancy interval births?

**Findings:**

This cohort study of 26 586 births to women enrolled in Emergency Medicaid found that when Medicaid expanded coverage to include prenatal care for these women, receipt of recommended prenatal care screenings and vaccines rapidly increased. Prenatal care coverage expansion was not associated with a meaningful improvement in postpartum contraceptive use or short interpregnancy interval births.

**Meaning:**

These findings suggest that expansion of prenatal care coverage, without postpartum care or ongoing coverage, improved receipt of evidence-based prenatal care, but guideline-concordant prenatal care alone was not sufficient to significantly improve other maternal and infant health outcomes among Emergency Medicaid recipients.

## Teaser

This cohort study examines extending prenatal care coverage and the association with postpartum contraception and short interpregnancy interval (IPI) births among women enrolled in Emergency Medicaid in Oregon and South Carolina.

## Introduction

An essential component of pregnancy care is the discussion of birth spacing recommendations and contraceptive counseling.^1^ The time interval between pregnancies is an important and modifiable risk factor for preventing future adverse birth outcomes.^2,3,4,5^ A large majority (74.4%) of pregnancies that occur within 18 months of birth are mistimed or unwanted.^3^ Postpartum contraception is a safe and effective strategy to prevent conception; however, barriers, particularly cost, exist to its correct and consistent use.^2,6^ Short interpregnancy intervals (IPI), or pregnancies that occur within 18 months of a prior delivery, may reflect a missed opportunity to promote reproductive justice, as well as maternal and infant health.^3,7^

Immigrant women with low income represent a uniquely high-risk group who are often unable to access prenatal and postpartum care.^8^ A critical source of coverage for these women is Medicaid, the largest single payer for obstetric care.^9^ Prenatal care is recognized as a priority preventive health service within the Medicaid program; however, this coverage does not extend to many immigrant women.^10^ By federal law, undocumented immigrants and documented immigrants who have been in the US for less than 5 years are not eligible for full-benefit (traditional) Medicaid.^8^ Instead, coverage for these women is limited to Emergency Medicaid, which restricts benefits to life-threatening conditions, including hospital admission for childbirth. No prenatal or postpartum care, including contraception, is covered.^8^

However, federal policy changes enacted in 2002 and 2009 gave states new options for Emergency Medicaid recipients to provide prenatal care coverage, regardless of their legal status or date of entry to the US.^10,11^ In 2002, the Children’s Health Insurance Program (CHIP) allowed states to use federal funding to designate the fetus as a low-income child and provide services on its behalf during pregnancy.^11^ The second policy option, the Children’s Health Insurance Program Reauthorization Act (CHIPRA), adopted in 2009, allowed states to drop the waiting requirement and receive federal funds to provide comprehensive coverage to immigrant women during the first 5 years of their legal residence. Oregon is 1 of 19 states that expanded prenatal care to the Emergency Medicaid population using the CHIP and CHIPRA policy options.^10^ However, comprehensive postpartum coverage was not included.

Earlier studies found increases in prenatal care and screenings following expanded coverage with CHIP and CHIPRA, but the impact on health outcomes varied.^10,12^ A study of birth certificate data found no association between the introduction of the CHIP and CHIPRA policies and the incidence of low birth weight, preterm birth, being small for gestational age, or infant death.^10^ However, this study was limited by reliance on birth certificate data, which could not isolate the population affected by the policy change. A second study in Oregon examined linked maternal and child Medicaid claims and found substantial improvements for infant health, but did not find substantial improvements in maternal health when prenatal care was expanded to the Emergency Medicaid population.^13^ This single-state study was limited by its reliance on Medicaid claims only.

This study aimed to determine the effect of a policy change expanding prenatal care to the Emergency Medicaid population. Our primary focus was the association of the policy change with subsequent receipt of postpartum contraception and short IPI births. We also examined a range of secondary outcomes to identify whether the policy was fully implemented (increases in the number of prenatal visits) and whether the policy change led to the receipt of guideline-concordant care (evidence-based recommendations for vaccinations and screenings) and improvements in infant health. We selected these outcomes based on the potential for prenatal care to influence the outcome and its ability to reliably be captured in claims and birth certificate data.^13^

## Methods

We conducted a retrospective cohort study using linked Medicaid claims and birth certificate data from Oregon and South Carolina from October 2010 to December 2018. To allow a minimum of 18 months of follow-up, we analyzed outcomes for births occurring from October 2010 until July 1, 2016. We compared Oregon with South Carolina, which did not expand coverage beyond the federal minimum. We included Oregon and South Carolina because both states have experienced similar growth in their immigrant population and have comparable immigrant populations in terms of size and country of origin.^15^ Data were obtained from both states’ Medicaid and Vital Statistics offices under a data use agreement. The study was approved by the Oregon Health & Science University institutional review board. Informed consent was not obtained; the data set did not contain identifiable information allowing participants to be identified and contacted to minimize loss of privacy or confidentiality. We followed the Strengthening the Reporting of Observational Studies in Epidemiology (STROBE) reporting guideline.^14^

Our study population was restricted to women, aged 12 to 44 years, enrolled in Emergency Medicaid, who gave birth in a hospital during our study period (eFigure in the Supplement). Consistent with previous studies, women with Emergency Medicaid were identified using program eligibility codes.^12^ Given that Emergency Medicaid covers only specific events, no additional enrollment restrictions in the program were applied.

During our study period, South Carolina did not cover prenatal care for Emergency Medicaid recipients, and neither state provided coverage for postpartum care recipients. Oregon rolled out prenatal care coverage for the Emergency Medicaid population on a county-by-county basis between 2008 and 2013. We excluded women with Emergency Medicaid with deliveries in counties that offered prenatal coverage before July 2011. This exclusion allowed us to study women with at least 9 months of data prior to and up to 60 months following the policy change in Oregon. We also excluded births with a gestational age reported less than 23 weeks or more than 44 weeks.

Our primary focus was determining whether expanding prenatal coverage improved receipt of postpartum contraception and reduced subsequent short IPI births. We hypothesized that prenatal care expansion might improve the use of postpartum contraception through improved health education and counseling on the availability of methods offered immediately postpartum. We defined postpartum contraception as the use of any method within 60 days of delivery. We classified postpartum contraception methods as sterilization, long-acting reversible contraception (LARC) (intrauterine devices [IUDs] and implants), and short-acting hormonal methods (injectables, oral contraception, patch, and ring). Sterilization, LARC, and injectable progestin were identified through inpatient or outpatient Medicaid procedure claims. Short-acting contraceptive methods were identified through the National Drug Code (NDC) in the Medicaid pharmacy claims file (eTable 1 in the Supplement).

We also examined the association of prenatal care coverage with subsequent short IPI births. We hypothesized that expanding prenatal care may reduce short IPI births by improving maternal health education or contraceptive counseling. Short IPI births were defined as less than 18 months between a birth and the date of conception of a subsequent pregnancy. We calculated the date of conception using either the date of last menses (Oregon) or gestational age (South Carolina) provided on the birth certificate. Births were identified using *International Classification of Diseases, Ninth Revision (ICD-9)*,^15^* International Statistical Classification of Diseases and Related Health Problems, Tenth Revision (ICD-10)*,^16^ and *Current Procedural Terminology (CPT) *codes.

We tested whether increased access to prenatal care was associated with changes in infant outcomes. We selected outcomes that could be reliably determined by birth certificate and claims data and that occurred proximate to the delivery. We examined premature birth and neonatal intensive care unit (NICU) admission. Premature birth was determined from the gestational age reported on the birth certificate form and defined as any birth occurring before 37 weeks’ gestation. NICU admissions were similarly identified through birth certificate data.

Finally, we assessed the association of prenatal care expansion on different measures of health care utilization and quality. We examined the number of prenatal care visits to assess whether women enrolled in Emergency Medicaid would attend prenatal care visits if they were covered. We sought to determine the quality of prenatal care provided to Emergency Medicaid recipients following the policy change by examining receipt of guideline-concordant services. We defined guideline-concordant care as the receipt of the following evidence-based services: prenatal visits, prenatal care screenings (anemia, blood type, group β *streptococcus*, syphilis, and ultrasound), and prenatal vaccinations (influenza). Prenatal visits were classified as the number of prenatal visits in Medicaid claims data. The codes used to identify the receipt of prenatal screening tests and influenza vaccinations are outlined in the eTable in the Supplement.

### Statistical Analysis

We abstracted demographic and clinical information from the birth certificate files and claims data. Prenatal care services (screening measures and vaccinations), maternal age, and zip code of residence were obtained from claims data. Zip codes were classified as rural using Rural-Urban Commuting Area Codes.^17^ From the birth certificate data, we obtained information on maternal race/ethnicity (which we collapsed into White, Black, Hispanic, and other/unknown), multiparity, multifetal gestations, body mass index (calculated as weight in kilograms divided by height in meters squared), history of previous cesarean birth, and pregnancy complications (preexisting or gestational diabetes or hypertension).^18^ Data on the birth certificate form, including race/ethnicity, were self-reported. These covariates, including race/ethnicity, were selected owing to literature suggesting they are potentially associated with our outcomes of interest.^3,4,6^ We captured mode of delivery for the current pregnancy from birth certificate data and corroborated this with claims data. History of cesarean birth was also available from birth certificate data.

We estimated the outcomes of Oregon’s prenatal coverage policy using a difference-in-differences analysis, exploiting differential implementation across Oregon counties during 3 separate periods (July 2011, April 2012, and October 2013). Of the 36 counties in Oregon, 7 had already implemented the policy before 2011 and were excluded from the study, 7 implemented the policy in July 2011, 1 implemented the policy in April 2012, and the remaining 21 counties implemented the policy in October 2013. To account for this staggered implementation, we used a difference-in-differences regression that estimated program outcomes by implementation wave and aggregated interventions and wave-specific estimates to summarize the overall outcome of the policy.^19^ This approach reflects advances in the literature demonstrating that estimates from the standard 2-way fixed-effects models may be biased in the presence of staggered treatment timing and heterogeneous treatment outcomes across cohorts.^20,21^ As in other difference-in-differences designs, the comparison groups controlled for secular changes in outcomes. We conducted 2-sided tests with an alpha level of .05. For secondary outcomes, we used a modified Bonferroni correction to account for multiple comparisons.^22^

We tested for parallel trends and did not find evidence of nonparallel trends, with 1 exception (postpartum contraceptive use) (eTable 2 in the Supplement). For the postpartum contraceptive outcome, trend estimates implied that the difference-in-differences estimate is conservative (eTable 2 in the Supplement). Standard errors were clustered at the county level. We included a missing category for observations missing maternal county of residence on vital statistics records. We modeled 12 estimates to capture both health outcomes (postpartum contraceptive use, short IPI births, preterm birth, NICU admissions) as well as measures of policy implementation (prenatal visits) and quality of prenatal care (guideline-concordant care). Covariates were selected for each model based on clinical or reported associations with the outcome of interest.^3,23,24^ Our analyses were conducted using R software version 4.0.3 (R Project for Statistical Computing) from August 2020 to March 2021.

## Results

The study population consisted of 26 586 births to women enrolled in Emergency Medicaid. Among these women, 14 749 (55.5%) were aged 25 to 35 years, 25 894 (97.4%) were Black, Hispanic, Native American, Alaskan, Pacific Islander, or Asian women or women with unknown race/ethnicity, and 17 905 (67.3%) lived in areas with urban zip codes. Table 1 displays demographic characteristics among women in the Oregon group (n = 12 013) and women in the South Carolina comparison group (n = 14 573). Compared with women in the Oregon group, women in South Carolina group were more likely to be aged younger than 20 years (924 women [6.3%] vs 612 women [5.1%]) and to have missing indicators of rural or urban status (2552 women [17.5%] vs 360 women [3.0%]). Women in the South Carolina group were less likely to identify as Hispanic (10 748 women [73.8%] vs 10 981 women [91.4%]), have a prior cesarean birth (2051 women [17.2%] vs 2409 women [20.1%]) and experience pregnancy comorbidities, including gestational diabetes (1113 women [7.6%] vs 1676 women [14.0%]) or prepregnancy diabetes (173 women [1.2%] vs 199 women [1.7%]), compared with women in Oregon.

**Table 1.** Demographics and Delivery Characteristics of Emergency Medicaid Births by State, 2010-2016

Maternal characteristics	Women, No. (%)	
	Treatment (Oregon)	Comparison (South Carolina)
No. (%)	12 013 (44.0)	14 573 (56.0)
Age, y		
<20	612 (5.1)	924 (6.3)
20-24	2428 (20.2)	3058 (21.0)
25-34	6383 (53.1)	8366 (57.4)
≥35	2590 (21.6)	2225 (15.3)
Race/ethnicity		
Non-Hispanic White	251 (2.1)	441 (3.0)
Non-Hispanic Black	71 (0.6)	228 (1.6)
Hispanic	10 981 (91.4)	10 748 (73.8)
Other^a^	710 (5.9)	3 156 (21.7)
Rurality		
Urban	8918 (74.2)	8987 (61.7)
Rural	2735 (22.8)	3034 (20.8)
Missing	360 (3.0)	2552 (17.5)
Body mass index, mean (SD)^b^	27.2 (5.45)	26.7 (5.36)
Multiparous	9651 (80.3)	11 053 (75.8)
Multifetal gestation	103 (0.9)	124 (0.9)
History of previous cesarean	2409 (20.1)	2501 (17.2)
Cesarean delivery, current pregnancy	3388 (28.2)	3999 (27.4)
Pregnancy comorbidities		
Hypertensive disorder of pregnancy	572 (4.8)	576 (4.0)
Chronic hypertension	160 (1.3)	112 (0.8)
Gestational diabetes	1676 (14.0)	1113 (7.6)
Preexisting diabetes	199 (1.7)	173 (1.2)

^a^ Other race/ethnicity included Native American, Alaskan, Pacific Islander, Asian, and unknown.

^b^ Body mass index calculated as weight in kilograms divided by weight in meters squared.

**Figure 1.**
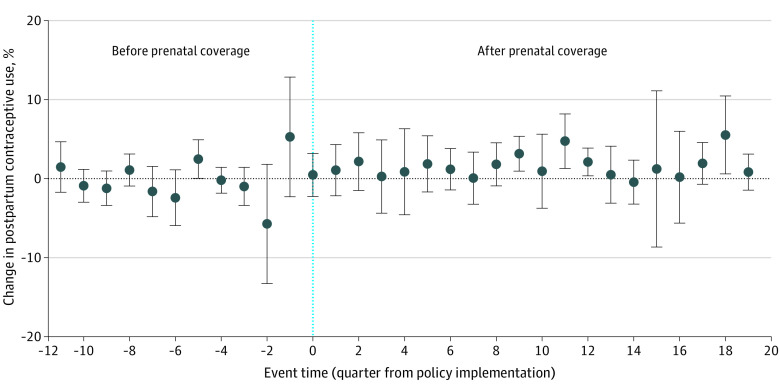
Adjusted Trend Estimates of Postpartum Contraceptive Use Among Emergency Medicaid Recipients in Oregon and South Carolina, 2010-2016 Estimates are adjusted for maternal age, rural location, cesarean birth, preterm birth, medical comorbidities of pregnancy (preexisting and gestational hypertension and diabetes). Vertical line denotes policy implementation. Whiskers denote 95% CIs.

**Figure 2.**
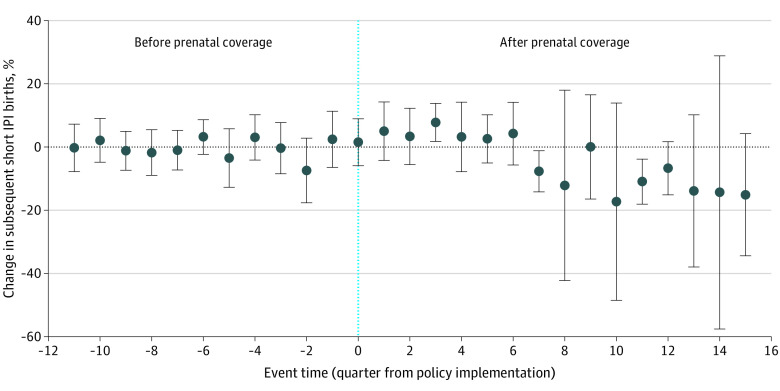
Adjusted Trend Estimates of Short Interpregnancy Interval (IPI) Births Among Emergency Medicaid Recipients in Oregon and South Carolina, 2010-2016 Estimates are adjusted for maternal age, rural location, cesarean birth, preterm birth, medical comorbidities of pregnancy (preexisting and gestational hypertension and diabetes). Vertical line denotes policy implementation. Whiskers denote 95% CIs.

Table 2 displays show changes in our primary outcomes before and after the policy across counties in Oregon. As previously noted, prenatal care expansion occurred in 3 waves across counties in Oregon. South Carolina did not implement this policy, but we display changes in outcomes for the early part of the study period (2011-2013) and the latter part (2014-2016) to indicate secular trends. Following the expansion of prenatal care in Oregon, there was a statistically significant increase in postpartum contraceptive use (1.5 percentage points [95% CI, 0.4 to 2.6 percentage points]) (Figure 1). There was no association between the introduction of prenatal care coverage and short IPI births (−4.5 percentage points [95% CI, −9.5 to 0.5 percentage points]) (Figure 2). Furthermore, expansion of prenatal care was not associated with a significant change in preterm birth (−0.6 percentage points [95% CI, −3.2 to 2.0 percentage points]) or NICU admission (0.8 percentage points [95% CI, −2.0 to 5.1 percentage points]) (Table 3).

**Table 2.** Changes in Primary Outcomes Among the Emergency Medicaid Population Following Expansion of Prenatal Care, 2010-2016

Primary outcomes	Women, No. (%)				
	Treatment (Oregon)		Comparison (South Carolina)		Difference-in-difference estimate, adjusted difference, percentage points (95% CI)
	Prepolicy (n = 5937)	Postpolicy (n = 6076)	2011-2013 (n = 7284)	2014-2016 (n = 7289)	
Postpartum contraceptive use^a^^,^^b^	8 (0.1)	116 (1.9)	189 (2.6)	215 (2.9)	1.5 (0.4 to 2.6)
Short IPI births^b^^,^^c^	665 (11.2)	435 (12.3)^d^	884 (12.1)	486 (11.6)^d^	−4.5 (−9.5 to 0.5)

Abbreviation: IPI, interpregnancy interval.

^a^ Defined as any method of contraception received within 60 days of delivery.

^b^ Estimates adjusted for maternal age, rural location, and medical comorbidities of pregnancy, preterm birth, and cesarean delivery.

^c^ Defined as less than 18 months between date of birth and conception of subsequent pregnancy.

^d^ Proportions for the postpolicy periods for short IPI births were calculated as a proportion of births between July 2014-2016 with a subsequent short IPI. Ns were as follows: Oregon: 3533; South Carolina: 4184.

**Table 3.** Changes in Secondary Outcomes Among the Emergency Medicaid Population Following Expansion of Prenatal Care, 2010-2016

Secondary outcomes^a^	Women, No. (%)				Difference-in-difference estimate, adjusted difference, percentage points (95% CI)
	Treatment (Oregon)		Comparison (South Carolina)		
	Prepolicy (n = 5937)	Postpolicy (n = 6076)	2011-2013 (n = 7284)	2014-2017 (n = 7289)	
Infant outcomes					
Preterm birth (<37 weeks)	426 (7.2)	481 (7.9)	545 (7.5)	588 (8.1)	−0.6 (−3.2 to 2.0)
Neonatal intensive care unit admission	152 (2.6)	206 (3.4)	316 (4.3)	350 (4.8)	0.8 (−2.0 to 3.6)
Policy implementation measure					
All prenatal visits, mean (SD), No.	0.3 (2.6)	11.1 (8.0)	0.5 (2.4)	1.7 (4.9)	10.3 (0.9)
Guideline-concordant care measures^b^					
Anemia	259 (4.4)	6784 (78.7)	252 (3.5)	614 (8.4)	65.7(54.2 to 77.1)
Blood type	43 (0.7)	1734 (28.5)	76 (1.0)	236 (3.2)	24.1 (11.7 to 36.4)
Obstetric panel	139 (2.3)	1547 (25.5)	8 (0.1)	29 (0.4)	24.0 (14.3 to 33.7)
Group β *streptococcus*	203 (3.4)	4074 (67.1)	43 (0.6)	96 (1.3)	55.3 (47.5 to 54.9)
Chlamydia/gonorrhea	145 (2.4)	3375 (55.5)	102 (1.4)	229 (3.1)	48.4 (42.0 to 54.9)
Syphilis	148 (2.5)	1682 (27.7)	34 (0.5)	68 (0.9)	25.3 (16.5 to 34.1)
Ultrasound	179 (3.0)	4813 (79.2)	194 (2.7)	777 (10.7)	63.7 (53.3 to 74.0)
Influenza vaccination	44 (0.7)	1947 (32.0)	8 (0.1)	46 (0.6)	31.9 (27.4 to 36.3)

^a^ Estimate adjusted for maternal age, rurality, and medical comorbidities of pregnancy.

^b^ Guideline-concordant measures are defined as receipt of screening or vaccination services at any time point during pregnancy.

In contrast, following prenatal care expansion to women in Emergency Medicaid, there were significant increases in mean (SD) prenatal visits (increase of 10.3 [0.9] visits) and improvements in prenatal quality (Table 3). All measures of prenatal screening and vaccination demonstrated substantial increases. The smallest change occurred in the obstetric panel (an increase of 24.0 percentage points [95% CI, 14.3 to 33.7 percentage points]); the largest change was observed in screening for anemia (an increase of 65.7 percentage points [95% CI, 54.2 to 77.1 percentage points]). The percentage of women with a prenatal influenza vaccination increased by 31.9 percentage points (95% CI, 27.4 to 36.3 percentage points).

## Discussion

Our findings contribute to a growing body of evidence on the individual health and system outcomes of restricting access to pregnancy care by citizenship status among low-income individuals. Using data from vital statistics and Medicaid claims from 2 states, we found that including prenatal care coverage for Emergency Medicaid slightly improved postpartum contraceptive use but was not sufficient to change other important measures of maternal and infant health (short IPI births, preterm birth, or NICU admission). These findings are not explained by incomplete policy implementation. When prenatal care became a covered benefit, we observed a significant increase in both utilization of services and receipt of guideline-concordant prenatal care.

Ensuring access to and utilization of guideline-concordant prenatal care is essential. For example, failure to screen for and appropriately treat women in pregnancy with rhesus negative blood type can lead to difficulty in obtaining timely transfusion services for women and can lead to fetal hydrops.^25^ Screening for group β *streptococcus* colonization in pregnancy and appropriate prophylaxis during labor can prevent the most common etiology of severe neonatal sepsis. A growing body of evidence demonstrates the multifactorial consequences that untreated anemia has on maternal health.^26,27,28,29^

Despite the improvements in these quality measures, we did not find an association between expansion of prenatal care and the majority of our health outcomes. We did find that prenatal care expansion was associated with a statistically significant improvement in postpartum contraceptive use among women covered by Emergency Medicaid (1.5 percentage points [95% CI, 0.4-2.6 percentage points]) This relatively modest increase is likely to provide individual benefits for the women receiving care. Still, it is unlikely to translate into significant population-level health benefits.

IPIs less than 18 months are associated with increased maternal mortality, severe morbidity in women older than 35 years, and increased risks of adverse fetal and infant outcomes in younger women.^30^ In our study, IPI decreased over time, but it did not appear to be associated with the introduction of Oregon’s expansion of prenatal care.^3^ This suggests that contraceptive education in the prenatal period alone is not sufficient to reduce short IPI births.

The US is facing a maternal mortality crisis that disproportionately affects low-income women and women from minority racial/ethnic groups.^31^ Medicaid covers obstetrical care for women who have low income and predominantly belong to minority racial/ethnic groups. Thus, it is critical to examine how Medicaid policy is associated with maternal health. Medicaid services for US citizens must extend for 60 days post partum for the woman. Recent research and advocacy efforts have highlighted that 60 days is inadequate. One-third of all maternal deaths occur in the first year postpartum and a majority are believed to be preventable; 60 days is insufficient time to prevent maternal mortality and severe morbidity.^32^ Structural factors, including racism and health care access, have been highlighted as contributing to the maternal health care crisis in the US, and advocates have introduced a range of bills and legislations to expand Medicaid coverage.^33^ Ongoing health coverage and care across an individual’s reproductive life span has been identified as critical to eliminating disparities in maternal health in the US.^32,33^ The Medicaid and CHIP Payment and Access Commission recently voted to recommend that Congress guarantee 12 months of postpartum care with federal funds for women enrolled in Medicaid and CHIP, an important step in advancing coverage.^34^ The Commission did not discuss the extension of postpartum coverage for women enrolled in Emergency Medicaid or by means of CHIP’s Unborn Child Clause.^34^

Scant attention has been paid to the role that Emergency Medicaid may play in perpetuating disparities in maternal health. Sixty days of postpartum coverage has been recognized by public health experts as inadequate for maternal health; benefits provided by Emergency Medicaid are even more restricted. Coverage ends the day a woman enrolled in Emergency Medicaid gives birth. Recipients of Emergency Medicaid largely belong to minority racial/ethnic groups, and a majority are Latina.^12^

### Limitations

Our study should be interpreted with the following limitations in mind. Our administrative data did not include information on documentation status, nativity, length of time in the US, hospital of delivery, or other factors that may influence prenatal care utilization and birth factors. Utilization of administration data means our databases are subject to errors in coding. However, our study is strengthened by the use of 2 distinct data sources, Medicaid claims and birth certificate data. Our study is subject to ascertainment bias: women with prenatal care coverage are more likely to be diagnosed with medical comorbidities due to increased exposure to the health care system. Our study used data from Oregon and South Carolina, which may affect our generalizability to other areas. We were not able to capture subsequent births to women who moved out of state or switched to a private payer. Thus, our measure of short IPI is a conservative estimate. Our study focused on the role of Medicaid coverage. We did not account for care received through charity programs, safety net clinics, or federally qualified health centers.

## Conclusions

Our study provides evidence that prenatal care expansion alone does not significantly improve selected markers of maternal or infant health. A meaningful reduction in maternal and infant health outcomes among the Emergency Medicaid population will most likely also require the inclusion of postpartum coverage and access to a choice of contraceptive methods.^35^ National dialogue on maternal health and Medicaid reform should not overlook the Emergency Medicaid program.

## Notes

Supplement. **eFigure.** Study Cohort Creation **eTable 1.** Outcome Measure Definitions by Coding System Type **eTable 2.** Trend Estimates for Primary and Secondary Outcomes
