# Supplementary material for: Association of Expanded Prenatal Care Coverage for Immigrant Women With Postpartum Contraception and Short Interpregnancy Interval Births
Source: JAMA Netw Open. 2021 Aug 2;4(8):e2118912. doi: 10.1001/jamanetworkopen.2021.18912 (PMC8329738; doi:10.1001/jamanetworkopen.2021.18912)
Supplement: Supplement. — eFigure. Study Cohort Creation eTable 1. Outcome Measure Definitions by Coding System Type eTable 2. Trend Estimates for Primary and Secondary Outcomes [file jamanetwopen-e2118912-s001.pdf]

Supplemental Online Content

Rodriguez MI, Kaufman M, Lindner S, Caughey AB, DeFede AL, McConnell KJ. Association of expanded prenatal care coverage for immigrant women with postpartum contraception and short interpregnancy interval births. *JAMA Netw Open.* 2021;4(8):e2118912. doi:10.1001/jamanetworkopen.2021.18912

eFigure. Study Cohort Creation

eTable 1. Outcome Measure Definitions by Coding System Type

eTable 2. Trend Estimates for Primary and Secondary Outcomes

This supplemental material has been provided by the authors to give readers additional information about their work.

© 2021 Rodriguez MI et al. *JAMA Network Open.*

eFigure. Study Cohort Creation

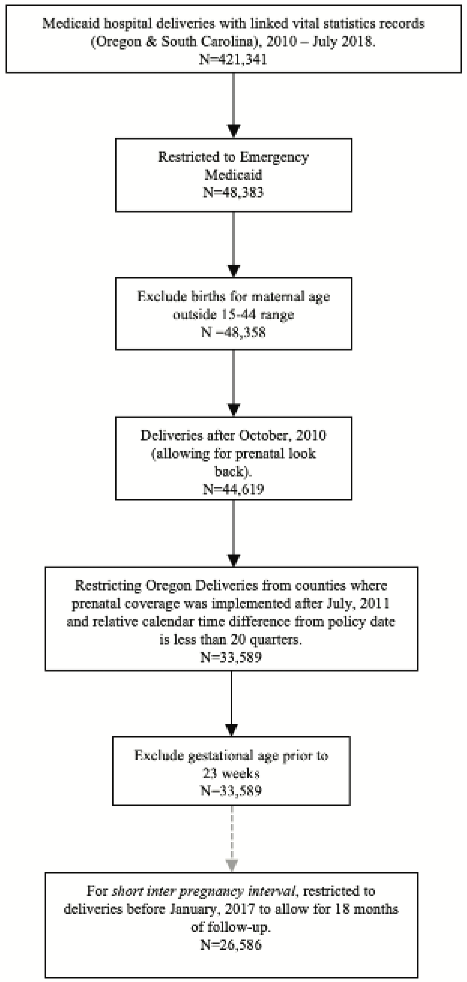

© 2021 Rodriguez MI et al. *JAMA Network Open.*

eTable 1. Outcome Measure Definitions by Coding System Type

Prenatal Care Measure	Codes
**Anemia Screening**	
CPT	80055; 85013; 85014; 85018;85025; 85027; 80081
**Blood Type Screening**	
CPT	86900; 86901
**Obstetrics Panel**	
CPT	80055; 80081
**Strep B Screening**	
CPT	87081
ICD-9	V286
ICD-10	Z3685
**Chlamydia/Gonorrhea Screening**	
CPT	87110; 87270; 87320; 87491; 87591
ICD-9	V745; 0980; 0982; 0994; 09940; 09941
ICD-10	A56; A54; A540; A5400; A5402; A5403; A5409; A541; A5424; A560; A5600; A5601; A5602; A5609; A561; A5611; A5619; A562; A563; A564; A568; A64; A71; A710; A711; A719; A748; A7481; A7489; A749; Z113; Z118; Z209; Z7189; Z7251;
**Glucose Tolerance Test**	
CPT	82947; 82951
**Syphilis Screening**	
CPT	80055; 86592; 86593; 86781
**Anatomy Ultrasound**	
CPT	76801; 76802; 76803; 76804; 76805; 76806; 76807; 76808; 76809; 76810; 76811; 76812; 76817
**Flu Vaccination**	
CPT	90630; 90653; 90654; 90655; 90656; 90657; 90658; 90661; 90662; 90672; 90673; 90674; 90685; 90686; 90687; 90689; 90756

© 2021 Rodriguez MI et al. *JAMA Network Open.*

eTable 2. Trend Estimates for Primary and Secondary Outcomes

**Primary Outcomes**			**Secondary Outcomes**										
			**Prenatal Screening Outcome Models**									**Infant Outcomes**	
	**Short IPI**	**Contraception < 60 days from delivery***	**Prenatal visits ***	**Anemia**	**Blood Type**	**Obstetric Panel**	**Group Beta Strep**	**Chlamydia/ Gonorrhea**	**Syphilis**	**Ultrasound**	**Prenatal Flu Vaccination**	**Preterm Birth (<37 weeks)**	**NICU Admissions**
**Linear trend test for Parallel Pre-trends**													
Estimate	-0.002	-0.003	-0.035	-0.005	-0.001	-0.002	-0.004	-0.002	-0.002	-0.002	0.000	0.002	0.000
P-value	0.3011	0.0213	0.0659	0.5094	0.0558	0.6153	0.5713	0.5176	0.7056	0.2678	0.3577	0.1442	0.8444
P-value adjusted for multiple comparisons of secondary outcomes			0.2556	0.6590	1.00	0.6138	1.00	1.00	1.00	1.00	1.00	1.00	1.00
**Difference-in-Difference p-value adjusted for multiple comparisons**			<0.00001	<0.00001	<0.00001	0.00000	<0.00001	<0.00001	0.00001	<0.00001	<0.00001	1.00	1.00

© 2021 Rodriguez MI et al. *JAMA Network Open.*
